# Amino acid synthesis deficiencies

**DOI:** 10.1007/s10545-017-0063-1

**Published:** 2017-06-26

**Authors:** T. J. de Koning

**Affiliations:** 1 Paediatrician for Inborn Errors of Metabolism, University of Groningen, University Medical Centre Groningen, Groningen, Netherlands; 2 Department of Genetics and Paediatrics, HPC CB50, P.O. Box 30001, 9700 RB Groningen, The Netherlands

## Abstract

In recent years the number of disorders known to affect amino acid synthesis has grown rapidly. Nor is it just the number of disorders that has increased: the associated clinical phenotypes have also expanded spectacularly, primarily due to the advances of next generation sequencing diagnostics. In contrast to the “classical” inborn errors of metabolism in catabolic pathways, in which elevated levels of metabolites are easily detected in body fluids, synthesis defects present with low values of metabolites or, confusingly, even completely normal levels of amino acids. This makes the biochemical diagnosis of this relatively new group of metabolic diseases challenging. Defects in the synthesis pathways of serine metabolism, glutamine, proline and, recently, asparagine have all been reported. Although these amino acid synthesis defects are in unrelated metabolic pathways, they do share many clinical features. In children the central nervous system is primarily affected, giving rise to (congenital) microcephaly, early onset seizures and varying degrees of mental disability. The brain abnormalities are accompanied by skin disorders such as cutis laxa in defects of proline synthesis, collodion-like skin and ichthyosis in serine deficiency, and necrolytic erythema in glutamine deficiency. Hypomyelination with accompanying loss of brain volume and gyration defects can be observed on brain MRI in all synthesis disorders. In adults with defects in serine or proline synthesis, spastic paraplegia and several forms of polyneuropathy with or without intellectual disability appear to be the major symptoms in these late-presenting forms of amino acid disorders. This review provides a comprehensive overview of the disorders in amino acid synthesis.

## Introduction

In recent years exciting developments have taken place in the discovery of disorders of amino acid synthesis. Several new disorders have been reported as well as new phenotypes for already known amino acid synthesis deficiencies, which is mainly due to next generation sequencing of cohorts of patients with similar clinical phenotypes. A comprehensive overview of these recent developments in amino acid synthesis deficiencies will be presented here.

We all are aware of the fact that amino acids are the building blocks for peptide and protein synthesis and that they perform important functions in intermediate metabolism. However, many amino acids have specific cellular functions of their own, in neurotransmission for instance, or energy metabolism and detoxification. Historically, the biochemical analysis of elevated levels of amino acids or their degradation products in body fluids has been the cornerstone of diagnosing inborn errors of metabolism. In 1996, Jaeken and colleagues reported, for the first time, defects in the synthesis pathway of the amino acid serine in children with severe neurological symptoms. Low levels of serine and (glycine) in plasma and cerebrospinal fluid (CSF) were the major diagnostic clues to indicate a serine deficiency disorder. Since then, defects in other amino acids synthesis pathways have been reported. The study of patients with these synthesis defects unravels new and unique functions of the amino acids involved, for instance in foetal development of the central nervous system or maintenance of the peripheral nervous system. We have also recently learned that these disorders can give rise to a whole spectrum of clinical symptoms varying from lethal developmental defects to late onset adult spastic paraparesis. The detection of amino acid synthesis deficiencies poses specific challenges to our biochemical diagnostic procedures because low values are easily missed, especially in milder phenotypes. Much to our surprise, in some disorders, the plasma or CSF concentrations of amino acids are non-informative, thus necessitating the use of sequencing techniques to confirm a clinical diagnosis.

Awareness of amino acid synthesis disorders is important because of the potential therapeutic consequences and the sometimes very narrow window of opportunity to alter the course of the disease and prevent neurological damage.

## Serine deficiency

Defects in the genes encoding the three enzymes of the L-serine synthetic pathway have been reported and, not surprisingly, they all give rise to similar clinical phenotypes. L-serine is synthesized from the glycolytic intermediate 3-phosphoglycerate via three enzymatic conversions. The enzymes involved are 3-phosphoglycerate dehydrogenase (3-PGDH, OMIM 606879), 3-phosphohydroxypyruvate aminotransferase (PSAT, OMIM 610936) and phosphoserine phosphatase (PSP, OMIM 172480).

When serine deficiency disorders were first reported in paediatric patients it appeared that there were some differences in phenotype between the three defects. However, with recent insights obtained through whole-exome sequencing, it is now obvious that it is not possible to discriminate the different gene defects on clinical grounds. Molecular defects in the genes encoding the three enzymes can present with identical phenotypes ranging from a severe lethal antenatal phenotype to a milder adult onset polyneuropathy phenotype. However, recognition of serine deficiency is important because good treatment results have been reported with L-serine therapy.

### Lethal serine deficiency phenotype (Neu-Laxova syndrome)

The severe and lethal serine deficiency phenotype was already known as Neu-Laxova syndrome (NLS). In 2014, two different groups published mutations in serine synthesis genes in patients with this Neu-Laxova syndrome (Acuna-Hidalgo et al 2014, Shaheen et al 2014). From this it was obvious that defects in all three genes can give rise to the same clinical phenotype.

NLS causes intrauterine or early postnatal death. Affected children present with dysmorphic features consisting of proptosis of the eyes, abnormal eyelids, microcephaly, small round mouth, extensive skeletal abnormalities with contractures and webbing of fingers and toes; skin abnormalities resembling a collodion-like skin, and multiple structural abnormalities of the central nervous system with neural tube defects, cortical dysplasia, enlarged ventricular spaces and structural abnormalities of the cerebellum. Defects in serine metabolism were detected through whole exome sequencing collaborations, and not through detection of low values of serine in plasma. Limited data are available on serine concentrations in body fluids in patients with NLS, but in my personal experience, plasma serine values can be very low (<30 μmol/L). No treatment with L-serine has been reported in this severe phenotype, but given the extensive and very early onset developmental defects, successful treatment seems doubtful.

The 3-PGDH knock out mouse published some years ago very much resembles the NLS phenotype and, so far, rescue of the severe developmental defects in mice with L-serine therapy has not been reported (Yoshida et al 2004).

### Infantile serine deficiency phenotype

The majority of children with serine deficiency suffer from the infantile phenotype, with phosphoglycerate dehydrogenase deficiency as the major cause. The first cases reported by Jaeken et al (1996) can also be classified within this phenotype. Many of the children were born after intrauterine growth retardation and presented with congenital microcephaly. After birth, intractable seizures developed within weeks to months, and little to no psychomotor development was observed once seizures are present. Children subsequently developed a severe spastic quadriplegia during the first years of life.

It is important to recognize that congenital microcephaly and seizures are not obligatory. Some children present first with severe psychomotor disability accompanied by secondary microcephaly and failure to thrive (Brassier et al 2016). One of the other symptoms regularly seen in infants is congenital cataracts (Tabatabaie et al 2010).

As in many other inborn errors of metabolism that gives rise to an encephalopathy, no specific seizure or EEG pattern is observed in serine deficiency and infantile spasms, tonic-clonic seizures, tonic, atonic, gelastic and myoclonic seizures have all been reported. The same is true for associated EEG abnormalities; both hypsarithmia and multifocal seizure activity evolving towards Lennox-Gastaut syndrome have been observed. Although the clinical seizures can respond rapidly to therapy (within weeks), normalization or improvement of EEG abnormalities may take much longer, in some cases as long as 6–12 months (de Koning et al 1998; Brassier et al 2016). Cranial MRI in patients with serine deficiency shows a profound decrease of cerebral white matter volume due to hypomyelination (de Koning et al 2000). Cerebellar abnormalities were observed on MRI in only one patient with PSAT deficiency (reported by Hart et al 2007). However, given the extensive cerebellar defects that can be observed in NLS, cerebellar abnormalities will likely be present in more patients with serine deficiency than reported so far.

### Juvenile serine deficiency phenotype

At present, only one family has been reported with a juvenile onset, much milder serine deficiency phenotype (Tabatabaie et al 2011). In two sibs, who acquired normal early developmental milestones but subsequently developed moderate developmental disability, atypical absence seizures began at school age. Microcephaly was not present in these patients, nor were there pyramidal signs. The patients were diagnosed as teenagers because of low values of serine in plasma and later also found in CSF. This diagnostic evaluation was done as part of a work-up for their developmental delay. At that time, one of the two sibs also had severe behavioural abnormalities and mood disturbances. Cranial MRI in both adolescents was normal without any signs of hypomyelination. The serine deficiency was caused by mutations in *PHGDH*.

With only these two patients reported in the literature, it is difficult to speculate on the expanded clinical phenotype that may yet occur in these milder juvenile onset forms of serine deficiency.

### Adult serine deficiency phenotype

Méneret et al (2012) reported an adult patient with serine deficiency also caused by 3-PGDH deficiency. This patient was diagnosed with mild mental disability and mild cerebellar ataxia in childhood, but developed a progressive polyneuropathy in adulthood. In addition, congenital cataract was operated in infancy, clearly demonstrating that overlapping symptoms between the different clinical serine deficiency phenotypes can occur in individual patients. Given the role of L-serine in myelin synthesis and the fact that defects in serine palmitoyltransferase are a well-known cause of polyneuropathy (hereditary sensory and autonomic neuropathy type 1, OMIM 605712 and 605,713), it is very likely that adult patients exist who suffer only from polyneuropathy and represent the very mild end of the spectrum of serine deficiency. I am aware of more adults with mild serine deficiency with predominant polyneuropathy, a phenotype likely to be underdiagnosed (unpublished data).

### Diagnosis

Remarkably, the biochemical abnormalities found in all patients reported with either the infantile, juvenile or adult phenotype were identical in plasma and CSF, and so a diagnosis of serine deficiency can be suspected from routine amino acid analysis in plasma and CSF at any age. The values in plasma and CSF were quite similar in all phenotypes and do not correlate with disease severity, meaning that the phenotype cannot be predicted from the results of amino acid analysis. Serine deficiency is more pronounced in CSF, and is not influenced by the absorption of amino acids from the diet, an important confounder of plasma amino acids analyses. Analysis of urine amino acids to diagnose serine deficiency is not helpful because, for still unclear reasons, amino acid excretion is normal in patients. While enzymatic assays are available for the three L-serine synthesis enzymes, enzyme testing is being replaced more and more by DNA diagnostics. This is due to problems in the availability of the substrate required for the 3-PGDH assay and the debated sensitivity of the other assays (Hart et al 2007).

### Treatment

Successful treatment with L-serine has been reported in patients with the infantile, juvenile and adult phenotypes.

Patients with infantile 3-PGDH deficiency can be treated with oral supplementation of a high dose of L-serine (500–700 mg/kg/day) and, for selected children with an insufficient response of their seizures to L-serine monotherapy, glycine (200–300 mg/kg/day) can be added to the treatment (de Koning et al 1998). Treatment with amino acids, in general, has a good effect on wellbeing and behaviour as well as on the patient’s seizure frequency. In some patients, the seizures will disappear and accompanying EEG abnormalities will resolve. In others, the seizure frequency significantly improves. Unfortunately, amino acid therapy does not have a positive effect on psychomotor development in symptomatic patients even when their seizures are responding. No progress, or only very limited progress, in psychomotor development has been observed during (long term) treatment follow-up (de Koning et al 2002; Brassier et al 2016; personal observations). However, treatment can be successful when amino acid therapy is initiated before symptoms arise, either as antenatal L-serine therapy given to the mother or as immediate postnatal therapy on the first day of life in still asymptomatic patients (de Koning et al 2004; Hart et al 2007).

Interestingly, patients with the juvenile and adult phenotype require much lower dosages of L-serine than young children do. In adolescents and adults good treatment results were obtained with 100–150 mg/kg/day L-serine without added glycine to the treatment. Dose finding studies have not been performed in adults but, in the patient reported by Méneret et al (2012), 80 mg/kg/day L-serine was insufficient to correct the biochemical abnormalities in CSF but correction did occur with 100-150 mg/kg/day.

It is unclear what the most appropriate age would be to switch from higher to lower doses of L-serine in children growing older and becoming adolescents. L-Serine treatment in the juvenile patients reported by Tabatabaie et al (2011) was initiated at 15 years of age, so it seems safe to adjust the dose of L-serine from around this age.

### Serine deficiency mimic and secondary serine deficiency

Interestingly, mutations in the gene encoding the major serine transporter *SLC1A4* (OMIM 600229) leading to a defect in the ASCT 1 transporter, give rise to symptoms similar to those seen in serine synthesis disorders. Srour et al (2015) reported two sibs with secondary microcephaly, psychomotor disability and spastic tetraplegia. Both sibs had a thin corpus callosum on MRI, which was associated with non-specific white matter abnormalities in one sib and delayed myelination in the other. One of the sibs was more severely affected than the other. Damseh et al (2015) reported nine patients with microcephaly, hypomyelination, developmental delay and, to a variable extent, seizures. Heimer et al (2015) reported more patients, who also presented with developmental disability, spasticity, microcephaly, seizures and hypomyelination with a thin corpus callosum on brain MRI. Surprisingly, plasma amino acid concentrations were found to be normal in all patients. CSF amino acids were analysed in only one patient and also found to be normal. Differentiation of *SLC1A4* mutations from serine synthesis disorders on clinical grounds appears difficult, although congenital microcephaly seems to be more frequent in patients with serine synthesis defects and cataracts have not been reported in ASCT1 transporter deficiency.

Very low levels of plasma serine and CSF serine have been observed in patients with disorders other than defects in the serine synthesis pathway. For instance, low serine values were observed in patients with disorders of folate metabolism, Menkes syndrome, complex 1 deficiency, and patients with overwhelming viral illness (Surtees et al 1997; Keularts et al 2010; personal observations). Reasons for the low serine concentrations in folate disorders are obvious given the role of L-serine in single carbon metabolism, but one can only speculate upon the mechanisms giving rise to low serine values in the other disorders.

## Glutamine deficiency

Glutamine deficiency (OMIM 610015) appears to be ultra-rare among the amino acid synthesis disorders. Since this disorder was first reported by Häberle et al 2005, only three patients in total have been described in the literature (Häberle et al 2005; Häberle et al 2011).

Glutamine is the most abundant amino acid in human plasma and CSF. It accounts for up to 20% of the total amino acid content in the human body. Similar to serine, glutamine is an important metabolite involved in maintaining nitrogen balance and cellular energy metabolism. However, glutamine synthetase also plays a key role in regulating the concentrations of ammonia and glutamate in brain tissues by converting these potential neurotoxic metabolites to glutamine.

The first two patients reported with glutamine deficiency were severely affected newborns who presented with respiratory insufficiency, hypotonia, absence of spontaneous movement and primitive reflexes (Häberle et al 2005). Both patients developed generalized seizures. One patient died after 2 days due to cardiac failure. The other patient died after 4 weeks and had, in addition, severe erythematous skin lesions with blistering and gastro-intestinal symptoms. Cranial MRI of both patients showed extensive brain abnormalities with cerebral and cerebellar atrophy and agyria. In one patient, multiple paraventricular cysts in frontal and temporal lobes were observed. In the other, a gyration pattern resembling that of preterm infants was combined with a small cerebellum, white matter changes and subependymal cysts (Häberle et al 2005).

A third patient with a somewhat milder phenotype presented with neonatal seizures and developed a chronic encephalopathy with intractable seizures resulting in severe psychomotor disability (Häberle et al 2011). The boy also suffered from necrolytic skin abnormalities although this appeared to be episodic and was not present in the newborn period. Cranial MRI revealed hypomyelination and a thin corpus callosum, findings that are also observed in other amino acid synthesis disorders. Importantly, this patient demonstrates that glutamine deficiency is not necessarily a lethal disorder and can give rise to an encephalopathy observed in other amino acid synthesis disorders as well as many other inborn errors of metabolism.

### Diagnosis

Routine amino acid analysis showed a striking deficiency of glutamine in plasma, CSF and urine (Table 1). In contrast, the concentrations of glutamate (the metabolite before the metabolic block) were normal, and only mild and variable hyperammonemia was observed. The enzymatic defect was confirmed in immortalized lymphocytes combined with mutation analysis of the glutamine synthetase gene. All mutations observed in the three patients affected the active site of the protein (Frieg et al 2016).

**Table 1:** Clinical and laboratory findings in amino acid synthesis disorders

Disorder	Clinical features	Biochemical /molecular diagnosis	Treatment	Response to treatment
**Serine deficiency (3-PGDH, PSAT, PSP)**	***Lethal phenotype (Neu-Laxova)***			
	Severe IUGR, multiple congenital abnormalities, dysmorfic features, contractures, ptyrigium, syndactyly, neural tube defects	CSF serine not reported CSF glycine not reported	not reported	not reported
		Plasma serine not reported Plasma glycine not reported		
	Skin: ichthyosis, collodion-like	Molecular testing in all patients		
	MRI: atrophy, vertriculomegaly, lissencephaly, cerebellar hypoplasia, absence vermis			
	***Infantile phenotype*** (congenital) microcephaly, IUGR, intractable seizures, severe psychomotor retardation, spastic quadriplegia, cataracts,	CSF serine 6–11 μmol/L Reference 30–75 CSF glycine 1-normal μmol/L Reference 2–10	500mg/kg/day L-serine 200mg/kg/day glycine	Control of seizures or significantly lowered frequency, improvement of wellbeing. Increased white matter volume. No to limited effect on psychomotor development.
	Skin: not reported			
	MRI: hypomyelination and delayed myelination, cerebellar abnormalities	Plasma serine 28–64 μmol/L, reference 85–235 Plasma glycine 128 μmol/L –normal, reference 145–420		*Antenatal or presymptomatic treatment:* prevention of neurological abnormalities
				Control of seizures, improvement of behaviour and school performance
		Urine not informative		
	***Juvenile phenotype*** Mild- moderate intellectual deficiency, absence seizures, behavioural problems		100-150mg L-serine /kg/day	
		CSF serine 9 μmol/L Reference 20–45 CSF glycine normal Reference 2–10 Plasma serine 58,63 μmol/L reference 60–185 Plasma glycine normal Reference 125–365		
	Skin: striae rubra			
	MRI: no abnormalities			
	***Adult phenotype***		120 mg L-serine /kg/day	Improvement in ADL activities
	Congenital cataract Psychomotor retardation Polyneuropathy, cerebellar ataxia and nystagmus	Urine not informative		
		CSF serine 13 μmol/L Reference 15–50 CSF glycine normal Reference 2–10 Plasma serine 33 μmol/L Reference 65–170 Plasma glycine 93 μmol/L Reference 120–445		
	Skin: not reported			
	MRI: no abnormalities			
	EMG: Charcot-Marie-Tooth type 2 polyneuropathy			
**Glutamine deficiency**	***Neonatal phenotype***			
	Respiratory insufficiency, hypotonia, neonatal seizures, cardiac failure, gastrointestinal symptoms	Plasma glutamine 2-6 μmol/L Reference 250–700 CSF glutamine 11–12 μmol/L reference 245–650 Urine glutamine ND-8	not possible	
	Skin: epidermal necrolysis			
	MRI: abnormal gyration, cortical and cerebellar atrophy, white matter abnormalities, germinolytic cysts			
	***Milder phenotype*** Severe psychomotor retardation, intractable seizures, spastic diplegia,		L-glutamine up to 1020 mg/kg/day	Improvement of alertness and EEG abnormalities. Partial correction of CNS glutamine deficiency
	Skin: necrolytic skin rash	Plasma glutamine 8–354 μmol/L reference 250–700 CSF glutamine 121 μmol/L reference 245–650		
	MRI: hypomyelination, cortical atrophy, thin corpus callosum			
**Proline disorders**				
**P5CS deficiency (** *ALDH18A1* **)**	***Infantile cutis laxa phenotype***			
	Microcephaly, progeroid features, mental retardation, hypotonia, seizures, movement disorders, joint laxicity, (intra uterine) growth retardation, cataract, corneal abnormalities	Decrease of proline, ornithine, citrulline and arginine can be found. Most patients no biochemical abnormalities Molecular testing preferred	L-ornithine therapy unsuccessful (2 sibs), L-arginine 150mg/kg/day (1 patient)	L-arginine resulted in progression of psychomotor development, correction of biochemical abnormalities and increase of brain creatine
	Skin: cutis laxa, wrinkly skin			
	MRI: hypomyelination, thin corpus callosum, cerebellar abnormalities, tortuosity of brain vessels, low creatine peak on MRS			
	***Adult phenotype***			
	Spastic paraparesis, psychomotor delay, cognitive defects, cataract, cyclic vomiting	Low plasma citrulline in dominant form of spastic paraparesis, low value of the total sum of plasma citrulline, ornithine, proline and arginine in recessive form Molecular testing preferred	not reported	
**PYCR1 deficiency**	Joint laxity, typical facial features, psychomotor retardation, osteopenia, intrauterine growth retardation, hypotonia, movement disorders. Similar symptoms as in P5CS deficiency, except for cataract and corneal abnormalities	No biochemical abnormalities Molecular testing	not reported	
	Skin: cutis laxa, wrinkly skin			
	MRI: thin or absent corpus callosum, white matter abnormalities			
**PYCR2 deficiency**	Progressive microcephaly, severe failure to thrive, profound psychomotor retardation, typical facial features, seizures, spastic tetraplegia, ataxia, movement disorders	No biochemical abnormalities Molecular testing	not reported	
	Skin: no abnormalities reported			
	MRI: hypomyelination, thin corpus callosum, progressive cortical atrophy, cerebellar atrophy, thin brainstem, ventriculomegaly MRS elevated lactate			
**Asparagine deficiency**	Progressive (congenital) microcephaly, intractable seizures, hypotonia, spastic quadriplegia, severe psychomotor retardation, hyperekplexia, diaphragmatic eventration	Inconsistent biochemical abnormalities, in some patients asparagine in plasma and CSF reported to be low Molecular testing	l-asparagine 20mg/kg/day	Worsening of seizures
	Skin: no abnormalities			
	MRI hypomyelination, delayed myelination, cortical and cerebellar atrophy, decreased size of pons, ventriculomegaly, simplified gyration,			

*ADL* activities of daily living, *CSF* cerebrospinal fluid, *CNS* central nervous system, *EMG* electromyography, *IUGR* intrauterine growth retardation, *MRI* magnetic resonance imaging, *MRS* magnetic resonance spectroscopy, *ND* not detectable, *P5CS* pyroline-5-carboxylate synthase, *PYCR1* pyroline-5-carboxylate reductase 1, *PYCR2* pyroline-5-carboxylate reductase 2

It should be noted that, in the patient with the mild phenotype, analysis of plasma amino acids at the age of 6 months demonstrated glutamine values just below the reference range, indicating that marginally low concentrations of glutamine should alert us to suspect this diagnosis (Häberle et al 2011).

Finally, low values of plasma glutamine can be associated with severe illness, in particular in children suffering from multiple organ failure in intensive care units (Ekmark et al 2015).

### Treatment

No treatment could be attempted in the two severely affected patients, but treatment was attempted in the third patient (Häberle et al 2012). This patient was given increasing doses of L-glutamine, up to 1020 mg/kg/day, that resulted in a correction of plasma glutamine but only minimal increase of glutamine values in CSF. Despite the fact that biochemical correction was incomplete, a clear improvement of his EEG abnormalities was observed with only minimal improvement in his clinical status. It seems as in serine deficiency that only early intervention will result in better clinical outcome.

Recently, also the synthesis of nicotinamide adenine dinucleotide (NAD) was found to be disturbed in this disorder (Hu et al 2015). The depletion of NAD in cells could be rescued by nicotineamide supplementation, and this finding may open up new possibilities for (combined) treatment in affected patients.

## Disorders of proline synthesis

The first family with a defect in proline synthesis was reported more than 15 years ago by Baumgartner et al (2000). Only recently, mainly due to advances in exome sequencing, many more patients and other defects in the proline synthesis pathway have been reported. The clinical spectrum of disorders of proline synthesis has expanded in a spectacular way. Because the majority of these patients were diagnosed using molecular techniques, the disorders are mostly known by their gene symbols and (usually) not classified according to their accompanying enzyme deficiencies. The defects in proline synthesis are pyroline-5-carboxylate synthase (PSCS or ALDH18A1) deficiency, pyroline-5-carboxylate reductase 1 (PYCR1) deficiency and pyroline-5-carboxylate reductase 2 (PYCR2) deficiency.

Most of the patients diagnosed with P5CS deficiency and PYCR1 deficiency suffered from cutis laxa, and these disorders are therefore classified as autosomal recessive cutis laxa syndromes (PSCS AR cutis laxa type 2b, type 3a and type3b, OMIM 138250). However, the discovery of children and adults with specific heterozygous mutations in *ALDH18A1* that lead to autosomal dominant forms of pyroline-5-carboxylate synthase deficiency (OMIM 616603) shifts our classical paradigm of autosomal recessive enzyme defects towards that of (de novo) autosomal dominant paediatric and adult-onset inborn errors of metabolism (Coutelier et al 2015; Fischer-Zirnsak et al 2015). Since many patients are now being identified through exome sequencing, these findings have important implications for our filtering strategies and the interpretation of next generation sequencing (NGS) results, not only for defects in proline synthesis but for the inborn errors of metabolism in general.

### Pyroline-5-carboxylate synthase deficiency (ALDH18A1)

As in serine deficiency, disorders of proline synthesis can result in a broad spectrum of symptoms ranging from severe neonatal forms to adult onset spastic paraplegia.

### Infantile cutis laxa phenotype

A deficiency in P5CS will result in a combined deficiency of L-proline, L-ornithine, L-citrulline and L-arginine, a disorder first reported by Baumgartner et al (2000). In this first family, P5CS deficiency was a slowly progressive neurodegenerative disorder affecting both the central and peripheral nervous system, combined with cataracts and extensive connective tissue involvement. However, the majority of patients with P5CS deficiency subsequently reported were shown to have a cutis laxa syndrome (Bicknell et al 2008, Skidmore et al 2011, Martinelli et al 2012, and others).

The main features in P5CS deficiency are microcephaly, cutis laxa, progeroid features, mental disability, hypotonia, seizures, joint laxicity, (intra uterine) growth retardation, and cataract and corneal abnormalities. In some patients, additional movement disorders such as tremor and dystonia were also present (Mohamed et al 2011; Zampatti et al 2012; Wolthuis et al 2014). On cranial MRI, hypomyelination with a thin corpus callosum can be found, sometimes with cerebellar abnormalities.

Patients with AR cutis laxa syndromes due to P5CS deficiency show many overlapping features with patients diagnosed with PYCR1 deficiency (discussed below). Patients with P5CS present more often with cataracts and corneal abnormalities, symptoms that are rare in PYCR1 deficiency and abnormalities of plasma amino acids can be observed in P5CS deficiency. The two disorders can only be discriminated by their ultrastructural abnormalities in skin biopsies.

Interestingly, Fischer-Zirnsak et al (2015) demonstrated that de novo dominant mutations in *P5CS* can also cause a similar cutis laxa phenotype. They reported eight paediatric patients with cutis laxa and de novo mutations in *ALDH18A1*. All mutations affected the same Arg138 residue of P5CS, affecting P5CS activity and leading to mislocalisation of the mitochondrial protein.

As discussed above, de novo dominant mutations are a challenge for our NGS data filtering strategies and our interpretation of NGS results. Since many of the enzymes we study in metabolic disease are also multimeric protein complexes, these observations are very relevant for other enzymes in which dominant mutations affecting dimerization can give rise to a clinical phenotype.

### Adult spastic paraparesis phenotype

Coutelier et al (2015) reported *ALDH18A1* mutations in adults with spastic paraparesis. Autosomal recessive mutations were associated with a complex spastic paraplegia combined with cognitive impairment. Surprisingly, heterozygous mutations in *ALDH18A1* were also found in families with autosomal dominant spastic paraplegia as well as in sporadic spastic paraplegia patients (suggestive of de novo mutations).

Psychomotor disability and cognitive defects were only seen in patients with the recessive form and biallelic mutations, whereas lower limb spasticity was the predominant clinical sign in patients with dominant mutations. Less frequent findings were cataracts, as observed in paediatric cases. The skin abnormalities including cutis laxa seen in children were not reported in any of the adult patients.

Panza et al (2016) showed that dominant mutations in *ALDH18A1* are the cause of spastic paraparesis type 9, a dominant spastic paraplegia associated with cataract and cyclic vomiting. Heterozygous mutations can cause this disorder because they act as dominant-negative mutations at specific sites, affecting dimerization of the enzyme complex and hence enzyme activity. This again supports the paradigm shift in our field towards (de novo) dominant inborn errors of metabolism associated with enzyme deficiencies.

### Diagnosis

Amino acid analysis in the first family reported with P5CS deficiency showed a combined deficiency of proline, arginine, citrulline and ornithine—an unusual combination of amino acids decreases. Low values of arginine, citrulline and ornithine are all observed in urea cycle defects and its related disorders, but the combination with low proline is only observed in this defect. Plasma ammonia was only mildly increased and decreased after meals in contrast to what happens in urea cycle disorders.

Surprisingly, in the majority of paediatric patients reported, no abnormalities were detected in any of the plasma amino acids, and it is poorly understood why the results of biochemical testing are inconsistent in P5CS deficiency. Timing of plasma sampling might be one explanation, another might be whether the mutations are in- or outside the active sites of the enzyme complex (Panza et al 2016).

The finding of a low creatine peak on brain MRS by Martinelli et al (2012) is very interesting and certainly could be of help in other patients too. Low creatine on MRS not only helps in establishing a diagnosis, but can also potentially be used to monitor treatment results.

In adults with the recessive form of P5CS deficiency, the sum of plasma citrulline, ornithine, proline and arginine was indicative of a deficiency (Coutelier et al 2015). In adults with the dominant spastic paraplegia, an important reduction in plasma citrulline was seen in all (4/4) individuals tested, and this reduction could potentially serve as a diagnostic marker for P5CS-related spastic paraplegia (Coutelier et al 2015).

As discussed above, many of the (paediatric) patients had normal plasma amino acids upon testing, and a clinical diagnosis is usually confirmed by mutation analysis. As stated before, it is important to realize that observations of (de novo) heterozygous mutations with dominant negative effects warrant careful interpretation of sequence results, particularly when NGS strategies are used, and that the presence of a de novo mutation favours a trio design of testing patient-parent trios. Confirmation of sequence results that are difficult to interpret is hampered by the absence of an accessible P5CS enzyme assay.

### Treatment

In the first report, patients were treated with oral L-ornithine. One patient was treated from the age of five years and the sib from 12 years. Disappointingly, this treatment with L-ornithine had no clinical effect. The patient reported by Martinelli et al (2012), received L-arginine with good effect. L-Arginine was given because a decreased creatine peak on MRS was observed and L-arginine therapy restored the low creatine on spectroscopy. With mental disability being an important feature in the paediatric patients with P5CS deficiency, this is an important finding. Unfortunately, no other patients treated with L-arginine have been reported so far. The findings of low citrulline in adults with the dominant spastic paraplegia opens up the possibility of supplementing with L-citrulline, but results of this therapy have not yet been reported (Coutelier et al 2015).

### Pyroline-5-carboxylate reductase 1 (PYCR1) deficiency

A defect in the gene encoding pyroline-5-carboxylate reductase 1 was first reported by Reversade et al (2009) in patients with cutis laxa and progeroid features. Mutations in *PYCR1* were found in 35 affected patients (from 22 families) with a phenotype classified as either de Barsy syndrome, wrinkly-skin syndrome or gerodermia osteodysplastica (OMIM 179035). In none of these patients were abnormal plasma concentrations of proline detected. In another large series of patients, Dimopoulou et al (2013) reported that PYCR1 deficiency can cause a broad clinical spectrum. Wrinkly skin, joint laxity, typical facial features, psychomotor disability, osteopenia, intrauterine growth retardation and hypotonia were the most frequently observed and consistent clinical symptoms. Patients can also suffer from movement disorders similar to those seen in P5CS deficiency, and paediatric patients with PYCR1 deficiency share many clinical symptoms that overlap with those in patients with P5CS deficiency.

In these large series of patients, mainly missense mutations were found in *PYCR1*, with splice site mutations being next, similar to what is seen in most inborn errors of metabolism. Dimopoulou et al (2013) reported a genotype-phenotype correlation and demonstrated that patients with mutations in the first two exons were clinically less severely affected and had no or very mild intellectual disability.

Adult phenotypes of PYCR1 deficiency likely exist and it is to be expected that the adult phenotype will resemble adult forms of P5CS deficiency.

The work of Reversade et al (2009) implicated mitochondrial dysfunction in PYCR1 deficiency and increased apoptosis as a possible pathogenic mechanism related to the disturbed intracellular proline synthesis. Such a role of PYCR1 in mitochondrial function was recently confirmed by others (Kuo et al 2016).

### Treatment

There are no reports of attempts to treat patients with PYCR1 deficiency with specific (amino acid) therapy.

### Diagnosis

Molecular testing is the primary method for diagnosing PYCR1 deficiency. Biochemical abnormalities were not found in any of the patients reported.

### Pyroline-5-carboxylate reductase 2 (PYCR2) deficiency

PYCR2 deficiency (OMIM 616420) was recently reported as a cause of secondary microcephaly and hypomyelination (Nakayama et al 2015). In two consanguineous families, one from Oman and one from Palestine, four affected children presented with developmental disability, failure to thrive and secondary microcephaly. Hypomyelination with a marked reduction of white matter volume was found on MRI along with a thin corpus callosum. Mutations in *PYCR2* were detected because the affected patients shared the same homozygous region, and a different homozygous missense mutation was found in each family. The disorder was classified as hypomyelinating leukodystrophy-10.

Another 18 patients from 11 families with *PYCR2* mutations were reported by Zaki et al (2016), expanding the clinical phenotype to that of an even more severe and lethal neurodegenerative disorder. In these patients, microcephaly, severe failure to thrive, profound psychomotor disability, typical facial features, ataxia and hyperkinetic movement disorders were observed, often in combination with spastic tetraplegia. Seizures were present in about half of the patients. The MRI findings in these patients were somewhat more variable, with progressive and extensive cortical atrophy as the main feature, and varying white matter abnormalities and thin corpus callosum in 60%. None of the reported patients survived beyond the age of 10 years, indicative of the severity and poor prognosis of PYCR2 deficiency.

Like PYCR1, PYCR2 is a mitochondrial protein. Zaki et al (2016) found evidence that PYCR2 is involved in regulating apoptosis under conditions of oxidative stress, and the protein likely plays a role in maintaining mitochondrial membrane potential. While mitochondrial dysfunction for PYCR1 had been demonstrated by Reversade et al (2009), a combined role for PYCR1 and PYCR2 in protection against oxidative stress was confirmed by Kuo et al (2016). This explains why mitochondrial dysfunction and increased lactate on brain MRS can be observed in PYCR2 deficiency (personal observations).

### Treatment

Similar to PYCR1 deficiency, there are no reports of treatment with amino acids in PYCR2 deficiency.

### Diagnosis

In patients with PYCR2 deficiency no biochemical abnormalities were observed. In particular, plasma proline was always normal. The only abnormal findings were marginal elevations of glutamate excretion in two patients tested for amino acid excretion in urine. Molecular testing is necessary to confirm a clinical diagnosis of PYCR2 deficiency.

## Asparagine deficiency

Similar to many of the patients with defects in proline synthesis, patients with a defect in asparagine were diagnosed through exome sequencing strategies. The first patients with asparagine deficiency (OMIM 615574) caused by defects in asparagine synthetase (*ASNS*) were reported by Ruzzo et al (2013). Of nine patients from four families, the majority had an epileptic encephalopathy with intractable seizures, progressive (congenital) microcephaly, severe developmental disability, axial hypotonia and spastic tetraplegia. As an additional clinical feature, acoustic startles and symptoms resembling hyperekplexia were observed in one of the families.

On MRI, decreased cerebral volume was seen in all patients, combined with a decreased size of the pons and a simplified gyration pattern in 6/9 patients. In some patients, progressive MRI abnormalities were noted on follow-up scans. As is not uncommon in epileptic encephalopathies associated with metabolic diseases, multiple seizure types and EEG abnormalities were present. All patients had missense mutations in the *ASNS* gene.

In 2015, Ben-Salem et al reported another child with congenital microcephaly who presented at day one with myoclonic seizures, did not acquire any developmental milestones and developed a severe spastic tetraplegia. He had pale optic discs and absence of visual responses. The MRI findings in this patient were more severely abnormal and also showed a decreased cerebral volume with atrophy of the corpus callosum and a marked ventriculomegaly. A third family with two affected sibs was described by Alfadhel et al (2015).

Seidahmed et al (2016) described two patients from unrelated families with excessive startles resembling hyperekplexia. The (acoustic) startles were striking clinical findings, including non-habituation of the head retraction reflex on glabellar and nose-tapping. In these two patients, truncal hypotonia was evident, which is in contrast with children with hyperekplexia who have generalized stiffness. Acoustic startles and non-habituation of the head-retraction reflex can provide important diagnostic clues to asparagine deficiency.

In patients for whom follow-up was reported, not only the clinical course and microcephaly, but also the abnormalities on MRI that were progressive. All these symptoms indicate that asparagine synthetase deficiency is a (slowly) progressive neurodegenerative disorder similar to PYCR2 deficiency (Palmer et al 2015).

A rapidly progressive course of disease has also been reported by Sun et al (2016) in two sibs who had, in addition, an eventration of the diaphragm resulting in severe respiratory complications. Although this may be a rare coincidental association, it was the first abnormality reported outside the central nervous system in this disorder and warrants careful investigation of multiple organ involvement.

Asparagine synthetase catalyses the transfer of ammonia from glutamine to aspartic acid to form asparagine, which is a ubiquitously expressed enzyme. Palmer et al (2015) found no correlation between the clinical phenotype and the effect of mutations on protein expression in model systems. It is puzzling how a ubiquitously expressed enzyme defect can affect the central nervous system in such a specific way, and we see a similar puzzling effect in the glutamine and proline synthesis disorders. Palmer et al (2015) showed in cultured skin fibroblasts of patients with asparagine deficiency that growth is restricted under conditions of limited availability of asparagine in the culture medium, and suggested that a similar mechanism may explain the central nervous system abnormalities.

### Diagnosis

Ruzzo et al (2013) commented on the potential pitfalls of a biochemical diagnosis in asparagine deficiency. Apart from our traditional focus on increased asparagine levels in catabolic amino acid disorders, asparagine levels are normally low in plasma and the lower range for asparagine in CSF is set at 0 mmol/l in many laboratories. In consequence, decreases in asparagine levels may not be noticed or picked up during the diagnostic process. However, low values of asparagine were indeed found in just over half of the families reported to date, with inconsistent elevations of glutamine. This means that while asparagine synthetase deficiency can be suspected on the basis of low values in plasma or CSF, it cannot be ruled out by normal values, making biochemical testing for this inborn error inferior to molecular testing.

### Treatment

Treatment was attempted in a single patient reported by Alrifai and Alfadhel (2016). In a 5.5-year-old boy, L-asparagine therapy resulted in minimal improvement of mental status/consciousness. However, it also led, surprisingly, to a worsening of his seizures, and the amino acid therapy was discontinued. The authors suggest that, similar to treatment in serine deficiency, only very early or prenatal therapy might result in prevention or amelioration of the neurological abnormalities. However, the authors warn not to draw firm conclusions from the results of treatment observed in this single case. Symptomatic treatment of the seizures appeared problematic in the reported cases, but Alrifai and Alfadhel (2016) found a favourable effect on the seizure frequency in their patient using high dose valproate.

## Conclusions

Defects in the synthesis pathways of the non-essential amino acids L-serine, L-glutamine, L-proline and L-asparagine predominantly affect the central nervous system, in particular in the infantile-onset forms. Interestingly, the synthesis defects have some clinical characteristics in common including microcephaly, psychomotor disability, seizures and white matter abnormalities on MRI. Defects in the synthesis of serine, glutamine and proline are also associated with specific symptoms of the skin, including collodion-like skin in serine deficiency, necrolytic erythema in glutamine deficiency and cutis laxa in proline synthesis disorders. We have learned in recent years that serine and proline deficiency disorders give rise to a broad spectrum of clinical phenotypes with a lethal (foetal) phenotype on one end and adult-onset neurological disease on the other.

In contrast to the spectacular gain of insight into the clinical spectrum we have witnessed in amino acids synthesis disorders, reports on how to treat these disorders are still rare, but promising in the serine synthesis disorders. Treatment should therefore gain more attention given the potential benefits of supplementing missing substrates. Whole exome sequencing has proven to be invaluable for elucidation of some of these disorders. Biochemical investigation, traditionally our first approach in patients with a suspected inborn error of metabolism, is insufficient, at least for diagnosing patients with proline and asparagine synthesis defects.

The observation that dominant mutations can also cause amino acid synthesis disorders will shift our classical paradigm of recessive inborn errors of metabolism. This shift requires careful interpretation of molecular diagnostics and the further development of functional assays for these amino acid disorders primarily approached by NGS techniques.
